# Tomographic Ultrasound and LED-Based Photoacoustic System for Preclinical Imaging

**DOI:** 10.3390/s20102793

**Published:** 2020-05-14

**Authors:** Kalloor Joseph Francis, Richell Booijink, Ruchi Bansal, Wiendelt Steenbergen

**Affiliations:** 1 Biomedical Photonics Imaging (BMPI), Technical Medical Center, University of Twente, 7522 NH Enschede, The Netherlands; 2 Translational Liver Research, Medical Cell BioPhysics (MCBP), Technical Medical Center, University of Twente, 7522 NH Enschede, The Netherlands; r.s.booijink@utwente.nl (R.B.); r.bansal@utwente.nl (R.B.)

**Keywords:** tomography, photoacoustic, ultrasound, small animal, liver, fibrosis

## Abstract

Small animals are widely used as disease models in medical research. Noninvasive imaging modalities with functional capability play an important role in studying the disease state and treatment progress. Photoacoustics, being a noninvasive and functional modality, has the potential for small-animal imaging. However, the conventional photoacoustic tomographic systems use pulsed lasers, making it expensive, bulky, and require long acquisition time. In this work, we propose the use of photoacoustic and ultrasound tomographic imaging with LEDs as the light source and acoustic detection using a linear transducer array. We have demonstrated full-view tomographic imaging of a euthanized mouse and a potential application in liver fibrosis research.

## 1. Introduction

Studying different diseases and developing new drugs in a controlled environment is vital in biomedical research. Small animals are widely used for this purpose, as they allow for controlled disease staging and evaluating the performance of drugs through histopathological validation [1]. Longitudinal monitoring of disease progression and treatment response to drugs can improve the outcome of preclinical studies and can reduce the number of laboratory animal deaths. Imaging modalities can be used for longitudinal monitoring of small animal models. However, there are limitations in using conventional imaging modalities such as MRI, CT, and ultrasound for small animal imaging [2]. Micro MRI is costly and has a slow data acquisition. Micro CT and PET, on the other hand, use ionizing radiation, which hinders longitudinal observations [2]. Ultrasound (US) is a noninvasive and real-time imaging modality but, being a structural imaging modality, in most cases it cannot quantify disease state. Photoacoustic (PA) imaging is a new modality that has functional and molecular capability while being noninvasive and real-time. Thus, PA is considered to be ideal for small animal imaging [3].

PA imaging utilizes pulsed light excitation to induce a temperature rise in optical absorbing structures inside the tissue resulting in thermoelastic expansion and acoustic wave generation. These acoustic waves are detected for imaging [4]. The advantage of PA imaging is that with optical excitation and acoustic detection it combines optical contrast at ultrasound resolution. Additionally, the use of ultrasound transducers enables us to combine PA imaging with conventional US imaging providing co-registered structural and functional imaging of the tissue. Several PA and US imaging systems were successfully demonstrated for small-animal whole-body imaging [3,5,6]. However, the use of a pulsed laser source in these systems not only makes them expensive but demands laser safe small animal labs, eye safety goggles, and additional manpower to operate the system. Therefore, for the wide use of PA imaging in small animal labs, there is a requirement for low cost, compact, safe to use tomographic systems which can be operated by a non-expert. Recent developments in LED-based PA imaging, being compact, low-cost, and safe to use, offer promising avenues to fill this gap [7]. LED-based handheld PA systems were used previously for small animal studies for imaging superficial structures such as tumors [8], wounds [9], and knee joints [7,10]. A limitation of the hand-held PA system using a linear transducer array is the limited view of the target tissue due to the directional sensitivity of the transducer. Additionally, with a small number of LED elements arranged on either side of the transducer, the imaging depth is shallow. We have recently developed a tomographic imaging configuration using a linear transducer array and four LED arrays, to overcome the limited view and to improve the imaging depth [11,12]. The system was originally developed for imaging finger joints for diagnosis and monitoring of rheumatoid arthritis [11].

In this study, we propose the application of our tomographic US and LED-based PA system for preclinical research. First, we demonstrate full-view tomographic imaging of the abdominal region of a mouse. We also compare the results with B-scan images obtained using a handheld probe. Further, we present a potential application of the system in liver fibrosis research. A large number of preclinical studies are currently being performed in small animals to develop antifibrotic therapies. However, the outcome of the preclinical study relies on endpoint histopathological analysis. A noninvasive imaging technique can provide longitudinal monitoring of animals and can improve the study outcome. We present the use of noninvasive and low-cost US and PA tomographic imaging system for liver imaging and compared the outcome with histology images.

## 2. Materials and Methods

An LED-based photoacoustic and ultrasound imaging system, AcousticX (Cyberdyne Inc., Japan), was used in this work. Four LED arrays having 576 elements (36 × 4 array) were used as the light source. We used LEDs having a wavelength of 850 nm, and each array has a pulse energy of 200 µJ with a pulse duration of 70 ns. We used a linear transducer array (128 elements) with a center frequency 7 MHz with 80% bandwidth for acoustic detection. A tomographic imaging configuration with four LED arrays and transducer scanning around the sample was used in this study.

**Figure 1:**
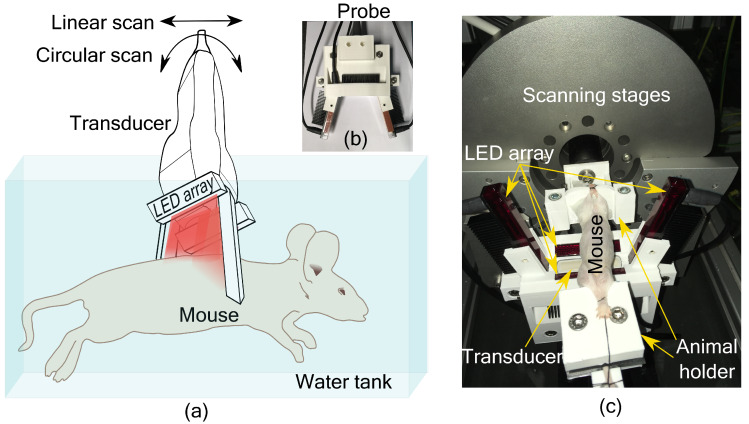
Small animal tomographic imaging system. (**a**) Schematic of the set-up with the imaging probe scanning around the mouse in a water tank. (**b**) Imaging probe with linear transducer array and four LED arrays in a 3D printed holder. (**c**) Photograph of the imaging set-up showing mouse holder, imaging probe and the scanning stages.

PA imaging using a linear transducer array suffers from low image quality due to incomplete acoustic detection. To overcome the limited view problem in linear array-based imaging, we recently developed a tomographic imaging configuration [11]. The system consists of an imaging probe with a linear transducer array and four LED arrays as shown in Figure 1a. Two LED arrays were placed parallel to the long axis of the transducer, geometrically pointing towards the focus of the transducer (20 mm depth). The other two LED arrays were placed on either side of the transducer along its short axis at an angle of 105° with the transducer surface. These two LED arrays were also placed at an angle of 5° with the imaging plane to minimize the acoustic reflections being detected. In this way, the object to be imaged is illuminated from three sides. The probe is then scanned around the object for tomographic imaging. Based on a simulation study, we have estimated that 16 angular views with an angular step of 22.5° is sufficient to form a full-view tomographic image [11]. The illumination configuration was also developed to provide a significant overlapping region between two angular scans. Interested readers can refer to the work in [11,12] for more details. A 3D printed holder encompassing the transducer and LED arrays to form the imaging probe is shown in Figure 1b. For small animal imaging, the probe was mounted on a motorized linear and circular scanning system, installed on top of a water tank as shown in Figure 1c. A 3D printed small animal holder was used to keep the mouse in place for imaging (Figure 1c).

Male Balb/c mice (8 weeks old) were used in this study. All the animals used in the study received ad libitum normal chow diet and normal water, and were housed with 12 h-light/12 h-dark cycle. All the animal experiments were carried out strictly according to the ethical guidelines for the Care and Use of Laboratory Animals (Utrecht University, The Netherlands). A proof-of-concept study was performed whereby liver fibrosis was induced in one mouse by a single injection of 1ml/kg of carbon tetrachloride (CCl_4_), and the control animal received olive oil. Mice were euthanized and imaged immediately using the PA and US tomographic system. The hair with the outer skin layer of the animals was removed around the abdominal region before imaging. After the imaging experiments, the livers of the animals were collected and fixed with cryomatrix in isopentane. Fixed livers were sectioned into 5 µm sections and immunohistological analysis using collagen I (fibrosis marker) antibody was performed.

Two scanning techniques were used in this study, involving a combination of circular and linear scans. To obtain a complete tomographic image, a circular scanning of the entire 360° was performed. Combined PA and US imaging were performed at each angular step. Typically, a circular scan takes 42.5 s. However, due to memory limitations of the system, the RF data was saved after each 180° scan and combined in the postprocessing stage. Multiple tomographic images of the abdominal area were obtained by translating the probe to a different location and repeating the circular scan. In the second experiment, the liver region was considered as the region of interest. Here, a limited view tomographic imaging was performed by spatial compounding of B-scan images from three angles (20° steps). In this case, linear scans were performed for the entire length (15 mm length) of the liver at each angular step. Each linear scan took 8.5 s and the 20° circular scan took 2.5 s. The US and PA B-scan images were formed by delay and sum algorithm. For speckle reduction and to improve the contrast of the US image, Bayesian mean filtering using an open source software was used [13]. To form tomographic images, the B-scan images from both US and PA imaging were rotated to the acquired angle and spatially compounded using a custom developed MATLAB program [14].

## 3. Results and Discussion

**Figure 2:**
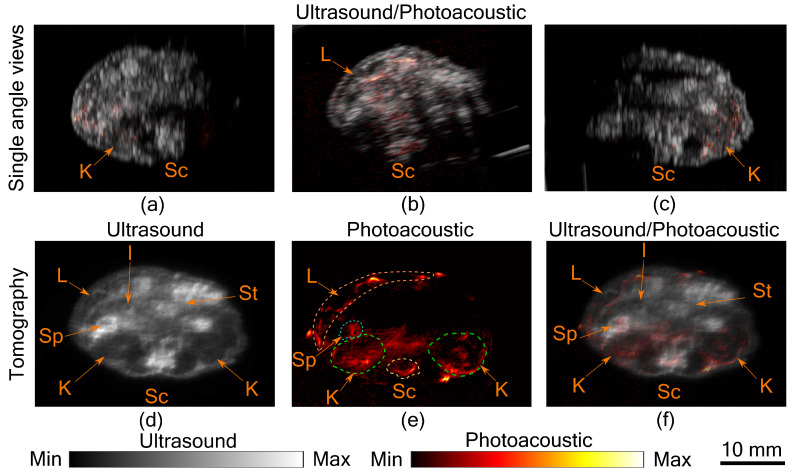
Tomographic photoacoustic and ultrasound imaging. (**a**–**c**) Co-registered ultrasound and photoacoustic B-Scan images of mouse abdomen, acquired from three different angles. Tomographic (**d**) ultrasound, (**e**) photoacoustic, and (**f**) co-registered ultrasound and photoacoustic image. Several organs in the abdominal region indicated by Sc-spinal cord, K-kidney, L-liver, Sp -spleen, St-stomach, and I-intestine. Dashed lines are used to mark the organs in the photoacoustic image.

In Figure 2a–c, co-registered ultrasound and photoacoustic B-scan images of a mouse abdomen, obtained from three different angles, have been presented. The images demonstrate the limited view problem in small animal imaging using a linear transducer array. The limited view arises from the directional sensitivity of the transducer resulting in structures oriented away from the transducer being undetected. The tomographic images obtained from spatial compounding of the B-scan images are shown in Figure 2d–f. The ultrasound image shows the capability of the tomographic system to image major abdominal organs such as the liver, kidney, spleen, stomach, and intestines. Additionally, anatomical structures such as skin and spinal cord are also imaged in this mode. Kidney and lobes of the liver can be seen as hypoechogenic regions, while the stomach, spleen, and intestine are visible as hyperechogenic regions. In PA images major blood vessels are visible beneath the skin and around the spinal cord. In addition to that, high PA signals were also observed from the liver, kidney, and spleen. For the imaging configuration used in this work, it was estimated that PA signal strength drops to 30% at a depth of 9.7 mm in soft tissue [11]. This finding also holds in this study, as most of the structures visible in PA images are within the 10 mm depth from the skin surface. Illumination from three sides and spatial compounding from several angles enable PA tomographic imaging to visualize most of the absorbing structures in the small animal. The abdominal diameter of Balb/c mice is mostly less than 30 mm and most of the organs of interest are within the 10 mm depth from the skin surface, making the system useful for small animal studies. However, there is indeed a region at the center where the signal to noise ratio is low, which can only be improved with better illumination methods. Another challenge in PA imaging is reflection artifacts from multiple structures inside the small animal body. There are several methods proposed to solve reflection artifacts in PA images [15]. Methods for improving the imaging depth and removing artifacts will be considered in the future. In this study, we have imaged a euthanized animal; however, imaging a live animal in this set-up is also possible by positioning the scanning stages such that the snout of the animal is above the water while the rest of the body immersed and/or using a breathing mask. Imaging speed is another factor that needs to be considered while imaging a live animal. The current scanning time is 42.5 s, which is longer when compared with the state-of-the-art system using a full ring transducer [3]. However, it is possible to synchronize the breathing cycle of the animal with the acquisition and by using postprocessing to reduce image distortion due to motion while acquisition [16].

**Figure 3:**
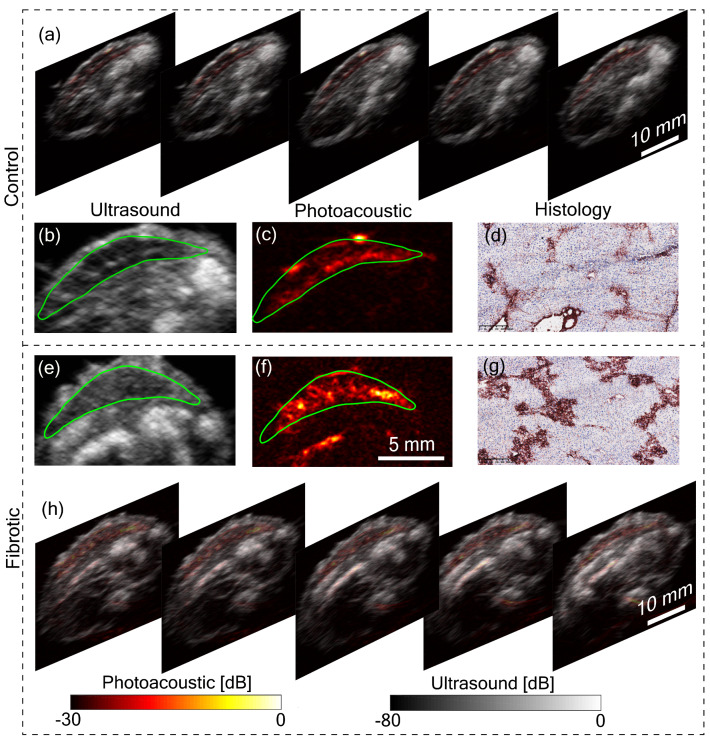
Tomographic imaging of the liver. (**a**) Tomographic photoacoustic and ultrasound images of a control mouse showing the liver at a scanning step of 2 mm. (**b**) Ultrasound image of the liver and (**c**) corresponding photoacoustic image. The green line markers the liver region. (**d**) Histology image of the control liver stained with Collagen I (fibrosis marker). (**e**) Ultrasound image of a mouse liver with fibrosis (CCl_4_-treated) and (**f**) corresponding photoacoustic image. (**g**) Histology image of the fibrotic liver stained with collagen I (fibrosis marker). (**h**) Tomographic photoacoustic and ultrasound images of the fibrotic mouse showing liver at a scanning step of 2 mm.

In Figure 2, the liver is visible in both US and PA. Being a superficial organ, small animal liver imaging is one of the potential applications where the proposed system can be used. To demonstrate this aspect, tomographic imaging of a control and a fibrotic mouse was performed. Figure 3a,h shows five tomographic slices of control and fibrotic mouse, respectively, with the liver in the top region of the image. Remarkable differences can be observed between control and fibrotic liver in both US and PA images. To analyze this difference, let us consider a pair of US and PA images of the liver from both control and fibrotic mouse shown in Figure 3b,c,e,f. The liver is marked with a green boundary. Two lobes of the liver are visible in the US images. In the US image, the difference in control and fibrotic liver is evident with the difference in echogenicity. Echogenicity can be characterized by the brightness level or the mean value of the region of interest. The mean pixel value was calculated with the liver as the region of interest. For the control animal, the calculated mean value was 0.13 ± 0.02, while it was 0.25 ± 0.03 for the fibrotic case. Additionally, heterogeneity computed using the variance was found to be 0.52 ± 0.11 for the control liver and 0.85 ± 0.10 for the fibrotic liver. These metrics were computed over the liver region with multiple tomographic slices stacked and normalized with the maximum value. The difference in structure evidenced in the US image is due to the hepatic nodularity developed due to scar formation in fibrotic liver [17]. In addition to the differences in US images, the PA images in Figure 3c,f show a difference in contrast between control and fibrotic liver. The contrast value computed for the liver region for the control animal was 0.48 ± 0.08 and 0.69 ± 0.08 for the fibrotic case. The primary reason for the high PA contrast in the fibrotic liver is due to the neovasculature in hypoxic liver cells [18]. Angiogenesis or neovascularization plays a crucial role in the progression of liver fibrosis and is also considered as an early indicator of fibrosis thus PA imaging can be used for monitoring the disease progression, while heterogeneity observed with US imaging reflects scar formation at a later stage of the disease [18]. The liver fibrosis was further confirmed using histopathological analysis. Figure 3d,g show histology images of control and fibrotic liver respectively with Collagen I immunostainings. The fibrotic liver (Figure 3g) shows an increased level of collagen bridges resulting from an accumulation of the extracellular matrix protein. These results are also in line with our previous small animal liver fibrosis study [19]. The main improvement we achieved in this work is the superior image quality from the tomographic system and the use of LED-based PA and US imaging. Further works will focus on including more animals in the study and developing a facility to image live animals.

The advantage of using an LED-based PA system is primarily the cost-effectiveness of the system. Nanosecond pulsed lasers and laser diodes are commonly used for tomographic imaging. The cost for a pulsed laser is approximately 70–200k$, and for laser diodes, with drivers, the cost ranges from 10–25k$. For LEDs with driving electronics, the cost is as lower as 10–15k$ [20]. Another benefit is that LED arrays can be integrated within the imaging probe, hence the system can be compact and suitable for small animal labs. Additionally, there is no requirement for a laser-safe imaging facility. However, the limitations are the low pulse energy resulting in low imaging depth, to an extent, this can be addressed using LED arrays with a large number of elements and frame averaging. The longer pulse duration (30–100 ns) achieved with driving circuits for LEDs is sufficient for stress confinement in tomographic imaging applications where the expected resolution is 1mm. However, unlike lasers, wavelength tuning is not possible with LED-based illumination. LED arrays with elements having multiple wavelengths were tested for oxygen saturation imaging compromising pulse energy and imaging depth. Importantly, for different imaging applications, a wide choice of LEDs is available ranging from visible to near-infrared wavelengths (470–980nm). Custom-developed power LED arrays arranged around the small animal along with ring US transducer can be an ideal configuration for small animal imagers in the future.

## 4. Conclusions

In this work, a tomographic ultrasound and LED-based photoacoustic system was demonstrated for small animal imaging. Results show that the tomographic ultrasound imaging can be used to distinguish major organs in the abdominal region of a small animal. The tomographic photoacoustic imaging is capable of imaging an approximate depth of 10 mm from the surface of the skin, allowing imaging of blood vessels and major organs. Limited imaging depth and artifacts are some of the hurdles for photoacoustic imaging in this proposed configuration. The applicability of the proposed imaging system is demonstrated in small animal liver imaging in a fibrotic mouse model. An increase in photoacoustic contrast and ultrasound echogenicity was observed in the fibrotic liver compared to that of the control mouse. The proposed tomographic ultrasound and photoacoustic imaging system offer a cost-effective, compact, and eye-safe alternative for the expensive laser-based system for small animal research.
